# Genomic analysis of GBS data reveals genes associated with facial pigmentation in Xinyang blue-shelled layers

**DOI:** 10.5194/aab-63-483-2020

**Published:** 2020-12-18

**Authors:** Haobin Hou, Xiaoliang Wang, Caiyun Zhang, Yingying Tu, Wenwei Lv, Xia Cai, Zhigang Xu, Junfeng Yao, Changsuo Yang

**Affiliations:** 1 Shanghai Academy of Agricultural Sciences, Shanghai 201106, China; 2 National Poultry Engineer Research Center, Shanghai 201106, China; 3 Shanghai Poultry Breeding Co., Ltd., Shanghai 201100, China

## Abstract

Facial pigmentation is an important economic trait of chickens, especially for laying hens, which will affect the carcass appearance of eliminated layers. Therefore, identifying the genomic regions and exploring the function of this region that contributes to understanding the variation of skin color traits is significant for breeding. In the study, 291 pure-line Xinyang blue-shelled laying hens were selected, of which 75 were dark-faced chickens and 216 were white-faced chickens. The population was sequenced and typed by GBS genotyping technology. The obtained high-quality SNPs and pigmentation phenotypes were analyzed by a genome-wide association study (GWAS) and a $F_{\mathrm{ST}}$ scan. Based on the two analytical methods, we identified a same genomic region (10.70–11.60 Mb) on chromosome 20 with 68 significant SNPs (-log⁡10(P)>6), mapped to 10 known genes, including *NPEPL1*, *EDN3*, *GNAS*, *C20orf85*, *VAPB*, *BMP7*, *TUBB1*, *ELMO2*, *DDX27*, and *NCOA5*, which are associated with dermal hyperpigmentation.

## 1 Introduction

Chicken skin color is mainly divided into three types: white, yellow, and black. Commercialized poultry varieties mostly have white or yellow skin traits. Some Chinese indigenous or Silkie chicken breeds have a black skin phenotype. These skin color traits are defined as quality traits and primarily controlled by genetic factors. It is one of the representative phenotypes of hypermelanization, which is caused by the deposition of numerous melanin particles in dermal and visceral tissue and known as fibromelanosis (FM) (Hutt, 1936). Melanocytes are the major cell types produced by birds and mammals to display body color. Melanocyte precursor cells, melanoblasts, are produced by the nerve crest and other progenitor cells in the early stage of embryogenesis (Bennett, 2006). Thought to originate in China and described by Marco Polo in the 13th century during his explorations of Asia, the poultry breeds with FM are believed to have been established well before the 13th century due to references in writings of unusual fowl in ancient Chinese (Haw, 2006). In 1911, researchers reported that the black trait in chicken is autosomal dominant (Bateson and Punnett, 1911). The physical location of FM (10.3–13.1 Mb, chromosome 20) was identified by a genome-wide single-nucleotide polymorphism (SNP)-trait association analysis (Dorshorst et al., 2010). Studies have shown that an inverted replication and junction of two genomic regions which are separated by more than 400 kb in wild-type individuals is the causal FM mutation. The mutation is formed by the inversion and replication of two genomic fragments Dup1 and Dup2 of 129 and 172 kb, respectively. The FM mutation has a dose effect on the regulation of black-skinned traits; the degree of blackness of the skin of homozygous FM mutants is significantly higher than that of heterozygous and wild-type homozygotes (Dorshorst et al., 2011; Shinomiya et al., 2012).

Chinese black-boned chicken breeds include Silkie, Dongxiang, Emei Black, Wumeng blacked-boned chicken, and other indigenous or hybrid breeds. These black-boned chicken breeds are characterized by the intensity of the black pigmentation in the skin, cockscombs, muscles, bones, and visceral organs, and it can be readily seen in the trachea, pericardium, blood vessels, sheaths of muscles and nerves, gonads, mesenteries of the gut, and periosteum of bone (Muroya et al., 2000). In particular, the Silkie is one of the most well-known breeds, and geneticists have researched this breed extensively as a pigmentation model. However, there are relatively few studies on the excavation and identification of pigmentation functional genes in indigenous blue-shelled chicken breeds. Recent advances in next-generation sequencing (Liao et al., 2015) have facilitated genome-wide SNP identification and SNP characterization. The advent of next-generation sequencing (NGS) has reduced the cost of genome sequencing to a level where genotyping by sequencing (GBS) is now considered a powerful tool for inquiring into a large number of genomic variations (SNPs). A large number of candidate genes related to different economic traits of livestock and poultry have been identified by this method, such as the coat color of sheep (Baazaoui et al., 2019), the fatty acid biosynthesis and litter traits of pigs (Kai et al., 2018; Wu et al., 2018), and the body weight of chicken (Fuwei et al., 2018). The combined analysis of genome-wide association study (GWAS) and selection signatures can effectively reveal agronomic traits of crops (You et al., 2018; Lu et al., 2019) and livestock economic traits (Yang et al., 2017). With advances in genomics tools, this study aims to identify SNP loci associated with dermal hyperpigmentation and useful SNP markers to track these loci; it also aims to determine new genetics donors through signatures of selection and associated loci, which will provide a theoretical basis for the selection of blue-shelled hens and the deposition of skin melanin.

## 2 Materials and methods

### 2.1 Experimental animals and phenotype

**Figure 1:**
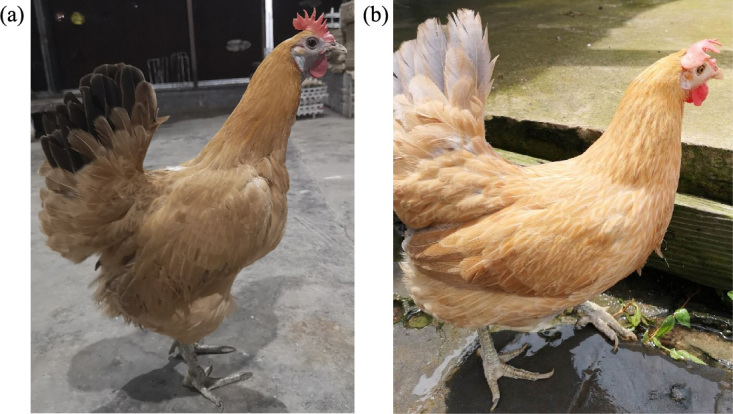
Xinyang blue-shelled pure-line layers: **(a)** dark-faced individuals and **(b)** white-faced individuals.

Animals used in this study were approved by the Ethics and Animal Welfare Committee of Shanghai Academy of Agricultural Sciences and performed according to the guidelines established by “the instructive notions with respect to caring for laboratory animals” issued by the Ministry of Science and Technology of the People's Republic of China (no. (2006)398). This study was conducted using 291 pure-line Xinyang blue-shelled layers (75 dark-faced (Fig. 1a) and 216 white-faced (Fig. 1b)) from Shanghai Poultry Breeding Co., Ltd., China. The original group of Xinyang blue-shelled layers comes from the local blue-shelled layers collected in Dongxiang County, Jiangxi Province. It is currently used as the parent line of the three-line matching for commercial egg production. The selected chicken population was from the same batch, with pedigree, single-cage feeding, and consistent management. Pulmonary venous blood collection and extraction of genomic DNA occurred at 36 weeks of age.

### 2.2 Library construction and sequencing

In the construction of the GBS library, the genome was digested by restriction endonucleases, and each sample was amplified by a bar-code interface. Then the samples were mixed, and the required fragments were selected to construct the library. Paired-end 150 sequencing was performed using an Illumina HiSeq sequencing platform.

### 2.3 Library inspection

After the construction of the library, the initial quantification was carried out with Qubit 2.0, and the library was diluted to 1 ng/µL. Then the insert size of the library was detected with Agilent 2100. After the insert size met expectations, the effective concentration of the library was quantified accurately by Q-PCR (library effective concentration >2 nM) to ensure the quality of the library.

### 2.4 Online sequencing

After the qualified library inspection, Illumina HiSeq PE150 was sequenced by pooling different libraries according to the effective concentration and quantity of target data. Efficient high-quality sequencing data were aligned to the reference genome by BWA (Li and Durbin, 2009) software (parameter: mem-t4-k32-M).

### 2.5 SNP detection and annotation

SNPs mainly refer to DNA sequence polymorphisms caused by variation in a single nucleotide at the genome level and include conversions and transversions of a single base. We used software such as SAMtools (Li et al., 2009) for group SNP detection. Bayesian models were used to detect polymorphic loci in the population. High-quality SNPs were obtained using ANNOVAR (Wang et al., 2010) software for population SNP annotations. ANNOVAR (Wang et al., 2010) is an efficient software tool that uses the latest information to functionally annotate genetic variants detected in multiple genomes.

### 2.6 Population principal component analysis

PCA only deals with autosomal data with the number of individuals n= XX, ignoring the higher than two allele loci and mismatch data. The analysis method of PCA is shown in the following equation:

1 $$d_{ik}^{\prime }=\left(d_{ik}-E\left(d_{ik}\right)\right)/\sqrt{E\left(d_{k}\right)\times \left(1-E(d_{k})/2\right)/2}$$

In Eq. (1), $d_{ik}$ represents the SNP of the individual i and k position. If the individual i is homozygous with the reference allele, then $d_{ik}=$0. If it is heterozygous, then $d_{ik}=1$. If the individual i and the non-reference allele are pure, $d_{ik}=2$. $E(d_{k})$ is the average value of $d_{k}$, and the individual sample covariance n×n matrix is calculated by X= MMT /S. GCTA (https://cnsgenomics.com/software/gcta/#Download, last access: 13 December 2020) software calculates feature vectors and feature values, and R software draws PCA distribution maps.

### 2.7 Association and selection signal analysis

In the process of GWAS analysis, the main factors causing false association are individual relationships and population stratification. Therefore, a mixed linear model was used to analyze the correlation of traits. Population genetic structure was used as a fixed effect, and an individual genetic relationship was used as a random effect to correct the effects of population structure and individual genetic relationships:

2 y=Xα+Zβ+Wμ+e,

where y is the phenotypic trait, **X** is the indicator matrix of the fixed effect, and α is the estimated parameter of the fixed effect; **Z** is the indicator matrix of the SNP, and β is the effect of the SNP; **W** is the indicator matrix of the random effect, and μ is the predicted random individual, and e is a random residual that obeys e∼ (0, $\delta_{e}^{2}$).

We considered dark and white face of chicken as case/control groups to obtain Wright's $F_{\mathrm{ST}}$ estimate for each SNP, using the $F_{\mathrm{ST}}$ option in PLINK 1.9 (Chang et al., 2015). In order to detect superior signals based on the allele frequency difference, markers were ranked according to raw $F_{\mathrm{ST}}$ values and plotted according to their genomic position based on the Gallus_gallus-5.0 assembly genome using R software (version 2.13.2). In addition, Haploview v4.2 software was used to analyze linkage disequilibrium and haplotype (Barrett et al., 2005).

### 2.8 Gene enrichment analysis

The gene contents in the candidate regions were annotated according to the outliers tested by $F_{\mathrm{ST}}$ from the Gallus_gallus-5.0 assembly genome. The Gene Ontology and Kyoto Encyclopedia of Genes and Genomes (KEGG) pathway analyses were performed with Metascape (http://metascape.org/gp/index.html#/main/step1, last access: 13 December 2020).

## 3 Results

### 3.1 Sequencing results

The total raw base sequence obtained after sequencing was 267.433 Gb, and the amount of individual sequencing was from 542.663 to 2079.192 Mb, with an average of 919.014 Mb per sample. The data obtained at this stage contained a certain amount of interference information, which will cause interference to subsequent analysis and need to be removed. According to the filtration conditions, strict quality control was obtained, and 267.402 Gb high-quality clean bases were obtained, with an average of 918.910 Mb per individual. The minimum sequencing of the individual was 542.510 Mb, and the maximum sequencing was 20795.560 Mb. The minimum value of Q20 was 93.29 %, indicating that the mass score of 93.29 % is greater than or equal to Q20 (error rate is less than 1 %), and the minimum value of Q30 is 85 %, indicating that the quality score of more than 85 % of bases is greater than Q30 (accurate). The rate was equal to 99.9 %, the sequencing quality was high, the GC distribution was normal, and the average value was 41.75 % (Supplement Table S1). All the samples used in the GBS library in this study were not contaminated, and the database was successfully constructed.

### 3.2 SNP detection and annotation

On comparing the 458 304 SNPs in this experiment with the chicken dbSNP library, the proportion of known and newly discovered SNPs was 88.9 % and 10.7 %, respectively. Because there is only chromosomal information in the reference dbSNP library, the information on the scaffold is missing. Therefore, a very small fraction (0.4 %) is on the scaffold (Fig. S1). Except for the SNPs distributed in the scaffold, the distribution of the remaining SNPs on each chromosome was analyzed. Most of the SNPs were distributed on chromosomes 1–5, while a small number were distributed on chromosomes 16, 32, W, and MT linkage groups (Fig. S2).

### 3.3 Population structure analysis

**Figure 2:**
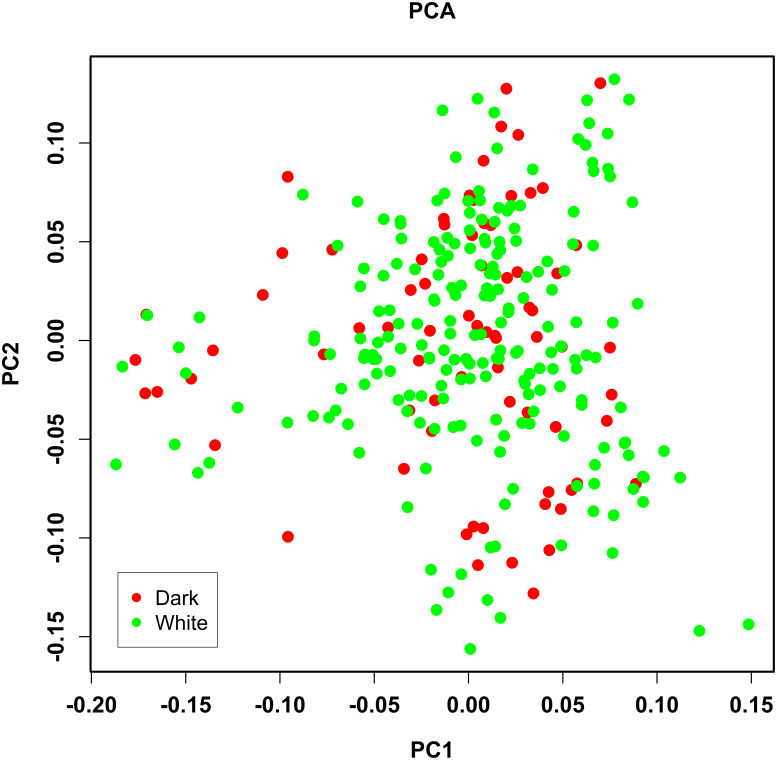
Population structure identified by the principal component analysis (PCA). Dark and white represent the face color of chickens.

PCA analysis of 458 304 independent SNPs revealed that the genetic distance of pure lines of dark- and white-faced Xinyang blue shell layers was close, and there was no obvious segregation, which was consistent with the breeding situation of the whole population (Fig. 2).

### 3.4 Genome-wide association analysis

**Figure 3:**
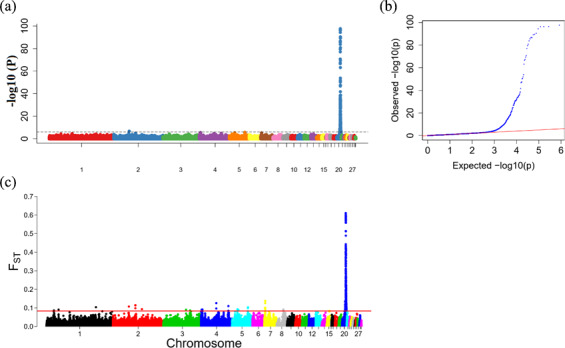
Results from the genome-wide association study (GWAS) of face color phenotype. **(a)** Manhattan plot of the GWAS for the face color phenotype; the y axis shows -log⁡10(P) values adjusted by a Bonferroni approach for the association tests, and the dashed horizontal line indicates the genome-wide significant threshold value (-log⁡10(P)>6). **(b)** The quantile–quantile plot of the P values; the x axis shows the expected -log⁡10(P) values, and the y axis shows the observed -log⁡10(P) values. **(c)** Genome-wide distribution of $F_{\mathrm{ST}}$ values for pairwise comparison of dark-faced vs. white-faced layers. The solid red line represents the threshold of significance (top 0.1 % SNPs).

**Figure 4:**
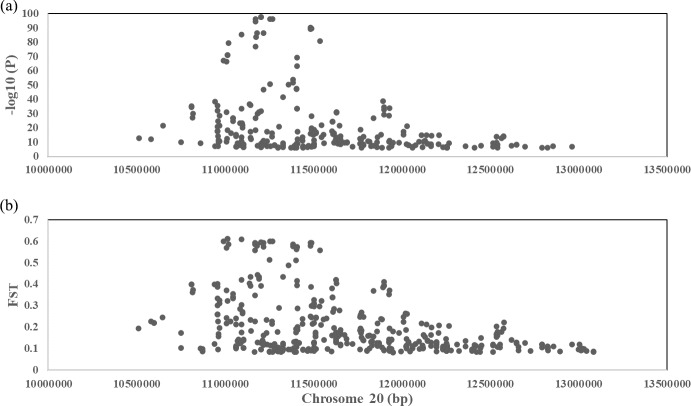
**(a)** A scatterplot presenting all the significant (-log⁡10(P)>6) single-nucleotide polymorphisms (SNPs) GWAS tested on chromosome 20 for the comb color phenotype. **(b)** A scatterplot presenting all the significant (top 0.1 %) single-nucleotide polymorphisms (SNPs) $F_{\mathrm{ST}}$ tested on chromosome 20 for the comb color phenotype.

**Figure 5:**
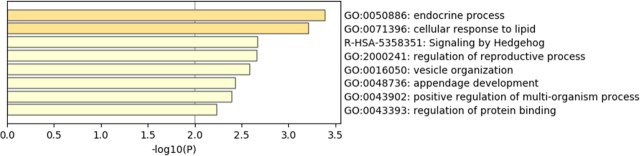
Enriched ontology clusters.

**Figure 6:**
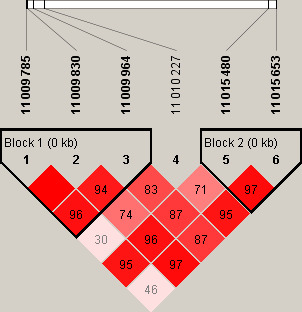
Linkage disequilibrium plot between six significant SNPs on *EDN3* gene.

To explore the genetic mechanism of face color in Xinyang blue-shelled chickens, we conducted a GWAS with the univariate method for face color phenotype. A total of 130 genome-wide significant SNPs located on chromosome 20 were successfully identified for the traits (Tables 1, S2, Figs. 3a, 4a). The associated SNPs located between regions 10.7 and 11.6 Mb were entirely attributable to a chromosomal region (∼ 0.9 Mb) harboring 10 genes including *EDN3* and *BMP7* (Table 1). The most significant SNP is at position 11 205 031 on chromosome 20. The results of detection $F_{\mathrm{ST}}$ revealed 187 potential SNPs distributed on same genomic regions with $F_{\mathrm{ST}}$ values ranging from 0.08 to 0.61 (Figs. 3c, 4b; Tables S3, 2). The 42 genes (Table S4) annotated by $F_{\mathrm{ST}}$ were analyzed for functional enrichment, and 31 significant biological processes were found (Table S5), which contain GO:0050886 (endocrine process) and GO:0071396 (cellular response to lipid) (Fig. 5). In addition, the linkage disequilibrium (LD) plot of six significant SNPs revealed two haplotypes (Block 1 and block 2) (Fig. 6).

## 4 Discussion

### 4.1 GBS assay for genome-wide SNP discovery

It was found that high-density SNPs are important for studying the genetic mechanism of complex traits or the mechanism of population genetics. After quality control, a total of 458 304 high-quality SNPs were identified in this study, more than in previous studies using this method in chickens (Liao et al., 2015; Pértille et al., 2016; Wang et al., 2017). If SNPs located on the linkage group are not considered, the high-quality SNPs identified in this study are mainly located on chromosomes 1 to 5, and the number of SNPs on chromosomes 6 to 10 is average, whereas a minimum number were found on chromosomes 16, 32, and W. This is the same as in a previous study, because chromosomes 1–5 are larger than the other chromosomes (Liao et al., 2015). After annotation, most of the SNPs are located in the intergenic and intron regions, and a very small number of SNPs are located in the exon functional region, indicating that only a few variant sites have an effect on protein translation. This result is the same as that reported in previous studies (Wang et al., 2017). In addition, 10.7 % of high-quality SNPs were found to be different from those in the dbSNP library, which cannot be achieved by SNP chips.

**Table 1:** Significant SNPs with extremely high P values revealed by a GWAS of face color phenotype on chromosome.

Significance	Peak	Reference	Alternative	-log⁡10	Peak	gene
Index	position (bp)			(P value)	effect	
13	11 205 031	C	T	97.6387925	intergenic	*NPEPL1, GNAS, STX16*
17	11 483 081	G	A	90.4384289	intronic	*C20orf85*,* ANKRD60*
11	11 094 964	C	T	85.54574511	upstream	*TUBB1, CTSZ, EDN3*
15	11 407 173	A	G	69.37487201	intronic	*APCDD1L, VAPB*
24	11 893 611	C	T	38.59275887	intronic	*BMP7, SPO11*
10	10 942 949	A	G	38.29324783	intronic	*ELMO2, SLC35C2, DDX27*

**Table 2:** Summary of the most interesting candidate genes within extreme signatures on chromosome 20.

Position (pb)	Markers	NMISS	$F_{\mathrm{ST}}$	Annotation
11 015 480	6	252	0.610881	*EDN3*
11 269 211	4	288	0.600183	*NPEPL1*
11 171 926	17	288	0.593077	*GNAS*
11 483 081	3	288	0.59301	*C20orf85*
11 407 173	20	245	0.573541	*VAPB*
11 093 783	3	282	0.420703	*TUBB1*
11 900 654	15	286	0.41139	*BMP7*
10 958 207	9	288	0.401988	*DDX27*
10 810 651	4	271	0.399717	*NCOA5*
10 942 949	2	288	0.398316	*ELMO2*

### 4.2 Genome-wide association analysis

The most significant genomic regions were identified, including *NPEPL1*, *EDN3*, *GNAS*, and *STX16* genes. Previous GWAS studies revealed that *GNAS*, *BMP7*, and *STX16* genes may be associated with hyperpigmentation of the visceral peritoneum (HVP) (Luo et al., 2013). The molecular function of *NPEPL1* is enriched to GO:0030145 (manganese ion binding), and the protein function is metallopeptidases. Endocrine process (GO:0050886) involves the secretion of or response to endocrine hormones. An endocrine hormone is a hormone released into the circulatory system. *CTSZ*, *EDN3*, and *GNAS* are involved this process. In poultry, hyperpigmentation is determined by a dominant autosomal gene conditionally termed Fm/*EDN3* (fibromelanosis) interacting with the sex-linked recessive wild type *id*${}^{+}$ gene and, possibly, the $W^{+}$ gene that determines the white color of the face. The phenotype is inherited in a Mendelian fashion with incomplete dominance (Shinomiya et al., 2012). Previous research and new gene terminology indicate that the Fm locus is on chromosome 20, with the *EDN3* gene as the main factor provoking fibromelanosis in Silkie chickens (Dorshorst et al., 2010, 2011).

The chickens used in the present study have been selected for over 19 generations and subjected to unified selection for egg quality and yield, and black face accounts for about a quarter of the population. In the present study, we also found some extremely significant SNP loci in the Fm locus region on chromosome 20. These SNPs were annotated, and regions related to dermal hyperpigmentation phenotypes were located, including the *EDN3* gene. *EDN3* is a gene with an acknowledged role in enhancing the proliferation of melanoblasts and melanocytic regulation (Baynash et al., 1994; Lahav et al., 1996; Dupin et al., 2000; Garcia et al., 2008; Chang et al., 2015). Endothelins are endothelium-derived vasoactive peptides involved in a variety of biological functions, and the active form of this protein is a 21 amino acid peptide processed from the precursor protein (Yanagisawa et al., 1988). The active peptide is a ligand for endothelin receptor type B (*EDNRB*) (Kurihara et al., 1999). The interaction this endothelin with *EDNRB* is essential for development of neural crest-derived cell lineages, such as melanocytes and enteric neurons (Baynash et al., 1994). Mutations in this gene and *EDNRB* have been associated with Hirschsprung disease (HSCR) (Gabriel et al., 2002) and Waardenburg syndrome (WS) (Amiel et al., 2008). The gene may also be related to pigmentation of sheep skin (Fariello et al., 2014; Baazaoui et al., 2019). Recently, a combined analysis of genomic and transcriptomic data suggests that the candidate gene related to the black-bone trait, *EDN3*, might interact with the upstream ncRNA *LOC101747896* to generate black skin color during melanogenesis (Li et al., 2020). The extremely significant SNP in this region can explain more than 90 % of whether the face of the Xinyang blue-shelled layer is dark or white. Research shows Cemani and Silkie have 2–4 times larger copy numbers than non-Fm chickens (Budhi et al., 2017). Such results have also been verified in two local chicken breeds in Sweden (Johansson et al., 2015). The size of these areas is over 100 kb and code for *EDN3*, *BMP7*, *TUBB1*, and *SLMO2*. *BMP7* plays a crucial role in regulate embryonic size (Engelman et al., 2006), renal function, and development (Gould et al., 2002; Zeisberg et al., 2003).

Recent studies have shown that a genomic region on chromosome 20 involving *EDN3* and *BMP7* is associated with hyperpigmentation in Dongxiang chicken comb (Dong et al., 2019). The results also showed that there was a significant correlation between melanin deposition and egg production, which suggests that the laying number of black comb layers is significantly higher than that of red comb chicken. Our research is also enriched in the GO:2000241 (regulation of reproductive process), involving *BMP7*, *AURKA*, *RTF2*, *EDN3*, and *SPO11* genes. However, we counted the number of laying eggs 129 d after 26 weeks of age, which show that there was no statistically significant effect between pigmentation and egg production, although white chickens have three more eggs on average than dark layers (Table S6). The red and dark comb of Dongxiang layers were two distinct lines, obviously different from our research population. However, the gene regions mapped are consistent with our results, from which it can be further inferred that dermal hyperpigmentation as a quality trait is determined by a few major genes. It has been established that the Fm mutation is positively correlated with the duplication of a segment that contains the *EDN3* gene (Dorshorst et al., 2011). The located gene regions of this study are consistent with the research, but the specific SNP sites are different. This suggests that the genetic mechanisms involved in different varieties or parts of chicken carcass (such as organs, comb, skin, and face) are also different and may be related to the adopted research methods. In the future, we need to further verify the gene expression in different tissues of chicken.

## 5 Conclusions

In this study, two analysis methods (GWAS and $F_{\mathrm{ST}}$) were used to reveal the major candidate region affecting chicken dermal hyperpigmentation, which was consistent with previous research results in other black-boned chicken breeds. This region contains *EDN3* gene, which is essential for development of melanocytes. It is also verified that a large number of effective molecular markers can be obtained by GBS technology in chicken, which can be used to correlate important economic traits of chicken and thus lay the foundation for maker-assisted selection or genome selection.

## Supplement

10.5194/aab-63-483-2020-supplement Table S1 contains sequencing reads, alignment statistics, and mean genome-wide coverage of each sample. Table S2 contains GWAS results of dermal hyperpigmentation in Xinyang blue-shelled pure-line layers. Table S3 contains the detection FST result of dermal hyperpigmentation in Xinyang blue-shelled pure-line layers. Table S4 contains the genes of enrichment analysis. Table S5 contains the results of GO analysis in Metascape. Table S6 contains the number of eggs laid in 129 d of Xinyang green-shell layer at 26 weeks of age. The supplement related to this article is available online at: https://doi.org/10.5194/aab-63-483-2020-supplement.

## Data Availability

The data generated by this project are included as supplementary information. Requests for the raw data should be made to the corresponding authors.
