# Supplementary material for: Genomic analysis of GBS data reveals genes associated with facial pigmentation in Xinyang blue-shelled layers
Source: Arch Anim Breed. 2020 Dec 18;63(2):483–91. doi: 10.5194/aab-63-483-2020 (PMC7810225; doi:10.5194/aab-63-483-2020)
Supplement: Table S1 contains sequencing reads, alignment statistics, and mean genome-wide coverage of each sample. Table S2 contains GWAS results of dermal hyperpigmentation in Xinyang blue-shelled pure-line layers. Table S3 contains the detection FST result of dermal hyperpigmentation in Xinyang blue-shelled  [file aab-63-483-supplement.zip › aab-63-483-2020-supplement-title-page.pdf]

Supplement of Arch. Anim. Breed., 63, 483–491, 2020
https://doi.org/10.5194/aab-63-483-2020-supplement
© Author(s) 2020. This work is distributed under
the Creative Commons Attribution 4.0 License.

Open Access
Archives Animal Breeding

Supplement of

Genomic analysis of GBS data reveals genes associated with facial pigmentation in Xinyang blue-shelled layers

Haobin Hou et al.

Correspondence to: Junfeng Yao (yaobison1983@163.com)

- aab-63-483-2020-supplement-title-page.pdf
- Supplement
  - Supplementary
    - Figure S1. The distribution of high-quality SNPs.jpg
    - Figure S2. The distributions for the SNPs.jpg
    - Table S1.xlsx
    - Table S2.xlsx
    - Table S3.xlsx
    - Table S4.xlsx
    - Table S5.xlsx
    - Table S6.xlsx

The copyright of individual parts of the supplement might differ from the CC BY 4.0 License.
