# Supplementary figures and images for: Genomic analysis of GBS data reveals genes associated with facial pigmentation in Xinyang blue-shelled layers
Source: Arch Anim Breed. 2020 Dec 18;63(2):483–91. doi: 10.5194/aab-63-483-2020 (PMC7810225; doi:10.5194/aab-63-483-2020)

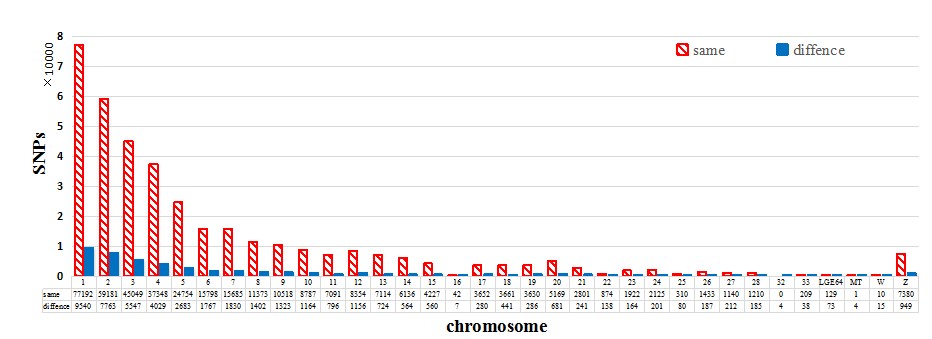

Supplement: Table S1 contains sequencing reads, alignment statistics, and mean genome-wide coverage of each sample. Table S2 contains GWAS results of dermal hyperpigmentation in Xinyang blue-shelled pure-line layers. Table S3 contains the detection FST result of dermal hyperpigmentation in Xinyang blue-shelled  [file aab-63-483-supplement.zip › Supplement/Supplementary/Figure S2. The distributions for the SNPs.jpg]

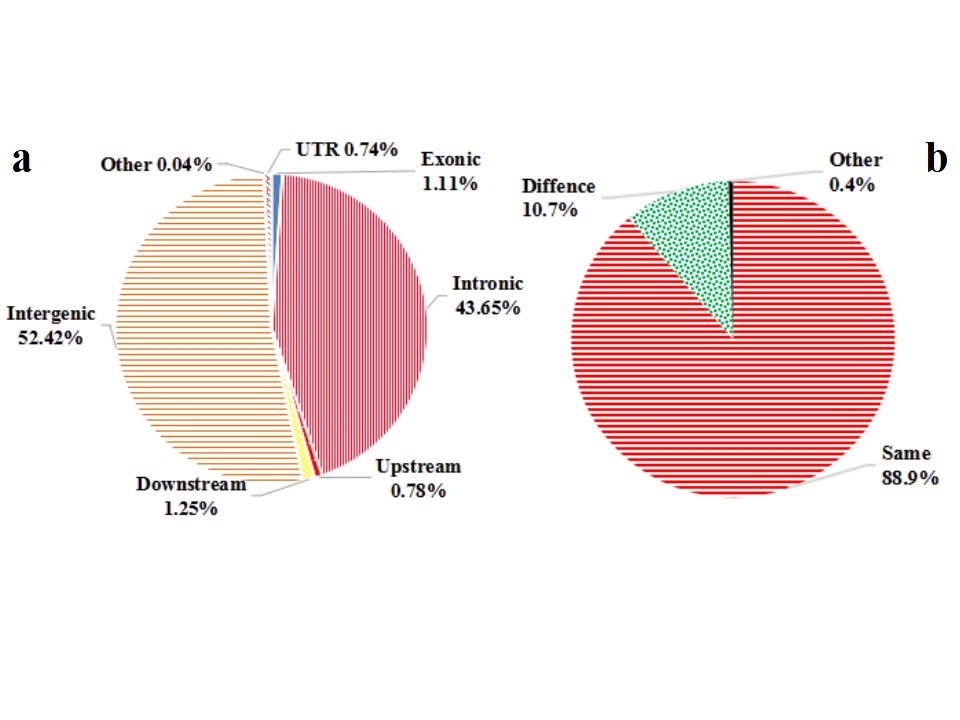

Supplement: Table S1 contains sequencing reads, alignment statistics, and mean genome-wide coverage of each sample. Table S2 contains GWAS results of dermal hyperpigmentation in Xinyang blue-shelled pure-line layers. Table S3 contains the detection FST result of dermal hyperpigmentation in Xinyang blue-shelled  [file aab-63-483-supplement.zip › Supplement/Supplementary/Figure S1. The distribution of high-quality SNPs.jpg]
